# Extracorporeal Testicular Ectopia through Inguinal Canal: A Case Report

**Published:** 2013-01-01

**Authors:** AM Haidar, BM Gharmool

**Affiliations:** Pediatric and Neonatal Surgical Department, Tripoli's Medical Center, Libya

**Keywords:** Neonate, Testicle, Ectopia

## Abstract

A preterm (36 week) neonate, presented with his left testicle hanging outside through the inguinal canal. The testicle was pexed in a sub-dartos pouch.

## INTRODUCTION

Testicular extrusion through the scrotal wall is called scrotoschisis. It is a rare entity and only a few cases have been reported [1-4]. We report a case of testicular extrusion through the inguinal canal in a preterm neonate.

## CASE REPORT

A 12-day-old male neonate presented with extrusion of left testicle from inguinal canal, at the level of superficial inguinal ring, since birth. He was born of an uneventful pregnancy and normal vaginal delivery at 36 weeks of gestation having birth weight of 1600gms. During examination, the left testicle, epididymis, and part of the cord was noted to be extruding through a small defect in the inguinal canal at the level of superficial inguinal ring, covered in a thick fibrotic layer resembling what we see occasionally in cases of gastroschisis (Fig. 1). The left scrotum was although empty but well formed and around the same size as the contralateral side that had right testicle inside. The baby was otherwise healthy. Laboratory investigations were normal, except for neonatal hypocalcaemia for which he received calcium gluconate before his presentation. The testicle was pexed after creating a sub-dartos pouch and ipsilateral inguinal exploration was done in which nothing abnormal was found. The patient was discharged the next day. During follow-up, the baby was thriving well, with left testicle palpable inside the scrotum.

**Figure:**
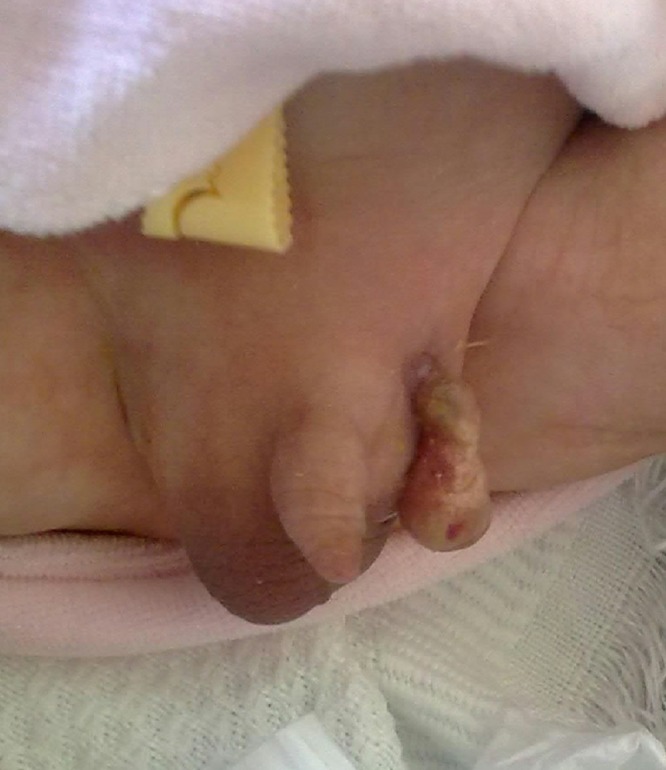
Figure 1: Testes lying outside the scrotum.

## DISCUSSION

In scrotoschisis, the testicular descent is in the normal pathway, but due to a defect in the scrotal skin wall, extrusion occurs, the cause of which is not fully understood yet. In our case, however, the testicular evisceration was through the inguinal canal which may not fit into the term scrotoschisis. This can be considered as extracorporeal testicular ectopia (ETE) or bubonoschisis, respective to the location of the defect. The usual sites of testicular ectopia are superficial inguinal pouch, femoral triangle, base of the penis, and perineum. Less than 10 cases of ETE through the scrotal defect have been reported in literature, 2 cases have been mentioned in association with a cesarean section delivery and were considered iatrogenic. Few cases were associated with meconium periorchitis. Most of the cases that have been reported were either bilateral or left sided [3-6]. We could not find any other case of ETE through the inguinal canal.

In general, ETE seems to affect otherwise healthy newborn males. The presentation is usually immediate and is not considered as an emergency as long as torsion is avoided by wrapping the extrusion in sterile wet gauze. Testicular torsion has been reported in a case of bilateral scrotoschisis [6]. Orchidopexy can be performed as soon as the patient is admitted and completed his preliminary workup. The immediate and short term prognosis is satisfactory, but long term follow up is needed to puberty to be able to comment on the long term complications, if any, do exist [4-6].

## Footnotes

**Source of Support:** Nil

**Conflict of Interest:** None
